# Traumatic Experience and Coping among Adolescent Refugees: A scoping review

**DOI:** 10.1007/s40653-025-00760-8

**Published:** 2025-09-04

**Authors:** Solomon D. Danga, Babatope O. Adebiyi, Erica Koegler, Conran Joseph, Nicolette V. Roman

**Affiliations:** 1 Center for Interdisciplinary Studies of Children, Families, and Society, University of the Western Cape, Private Bag x17, Bellville, 7535 South Africa; 2 School of Social Work, University of Missouri, St. Louis, MO 63121 USA; 3 Division of Physiotherapy, Faculty of Medicine and Health Sciences, Stellenbosch University, Tygerberg, South Africa

**Keywords:** Trauma, Coping strategies, Adolescent refugees, Mental health, Psychosocial adjustment

## Abstract

**Background:**

Adolescent refugees may be uniquely impacted by potential traumatic experiences due to their incomplete bio-psychosocial and cognitive development, dependence, and underdeveloped coping skills. Despite this vulnerability, there is a lack of clarity in the literature on the coping strategies adolescent refugees employ following trauma exposure and how these strategies are associated with their adjustment. The objective of this scoping review was to systematically identify the types of coping strategies used by adolescent refugees and examine the associations between trauma exposure and coping mechanisms.

**Methods:**

A comprehensive search of four electronic databases (Ebsco Host, PubMed, Scopus, and Web of Science) was conducted to identify relevant peer-reviewed articles. Inclusion criteria for studies were: 1) focused on the relationship between trauma and coping strategies was explicitly examined and discussed; 2) trauma was the primary predictor variable and the main focus of the study; 3) coping strategies were analyzed as outcome variables; 4) focused on adolescent refugees or asylum seekers aged 12–18 years as participants, including all genders; 5) articles were published in peer-reviewed journals between January 1, 2001, and June 20, 2021; 6) articles were written in English.

**Results:**

A total of 389 articles were identified as potentially relevant for the study, 6 articles were included in this scoping review. In total, 1694 participants were included across the included studies. Five included studies utilised a cross-sectional research design, and one study employed a case study. The review found that adolescent refugees mostly employed emotion-focused, avoidant and social support/ support-seeking coping strategies among the participants of the included studies. The majority of the included studies showed that traumatic experiences are more strongly associated with emotion-focused, avoidant and social support coping strategies than active and problem-focused strategies across diverse adolescent refugee populations. Avoidant-coping strategy was associated with maladjustment in young refugees.

**Conclusions:**

This scoping review suggests that future efforts should focus on equipping adolescent refugees with problem-solving and active coping strategies while addressing their psychosocial, cultural, and educational challenges to foster resilience and positive adjustment.

## Introduction

Forced displacement due to war, persecution, and human rights violations has led to a significant global increase in the number of refugees (UNHCR, 2021). Refugees are people living outside of their country who cannot return due to a well-founded fear of persecution because of their race, religion, nationality, political opinion, or membership in a particular social group (UNHCR, (United 1951)). In contrast, immigrants are voluntarily relocating to another country for economic, educational, or personal reasons. Additionally, asylum-seekers leave their country and seek protection in another country because they fear persecution or human rights violation. Still, they have not yet been legally recognised as a refugee and waiting to receive a decision on their asylum claim (Nickerson et al., 2017).

Refugees commonly experience stressful events because of political and religious oppression, being ethnic minorities, war, migration, and resettlement. Children and adolescent refugees migrate with histories of exposure to trauma (Lau & Thomas, 2008). Such trauma may include the violent death of a parent, injury to or torture of a family member, separation from parents, the disappearance of loved ones, enduring political oppression, deprivation of human rights and education, witnessing murder or massacre, exposure to bombardments, terrorist attack, forcible eviction from home, and detention (Kaplin et al., 2019; Khamis, 2021; Lau & Thomas, 2008). Young refugees may also experience physical injury and disability inflicted by violence, sexual assault, and subjection to child-soldier activities (UNHCR, 2017). Refugee adolescents are especially vulnerable to these traumatic events due to their incomplete bio-psychosocial and cognitive development, dependence, and underdeveloped coping skills (Lau & Thomas, 2008).

Previous studies documented that exposure to traumatic events significantly impacts young refugees'well-being and integration into a new country (Fazel & Stein, 2003; Murray et al., 2008). Additionally, refugees commonly face different adaptive challenges after resettlement while adjusting to a new society, language, social networks and cultural context (Markova & Sandal, 2016). However, despite adverse life events, most refugee youth eventually recover from their psychological distress and adapt well to the host society (Braun-Lewensohn & Al-Sayed, 2018; Lau & Thomas, 2008). Furthermore, studies have identified protective factors for children and adolescents, including responding to new situations, strong parental and social support, personal and community resources, positive self-esteem, and good temperament (Almqvist & Broberg, 1999; Braun-Lewensohn, 2015). Thus, one psychological construct that can influence well-being and adaptation is coping(Schneiderman et al., 2005). It is a crucial protective factor in adapting to the demands of the new resettlement environment (Dumont & Provost, 1999; Schneiderman et al., 2005).

Coping refers to individuals'thoughts and behaviour to manage stressful events'external and internal demands (Lazarus & Folkman, 1984). Lazarus and Folkman (1984) classified coping strategies into two different types: problem-focused and emotion-focused coping. Problem-focused coping aims to manage or modify the problem-generating discomfort, dealing with the stressor in different ways, such as planning actions or seeking information. On the contrary, emotion-focused coping attempts to regulate the emotional response to the problem through methods like looking for support. Coping behaviour has also been classified into four types, those who (i) are more engaged utilise approach-oriented coping, which involves direct problem-solving and taking action to increase understanding of the problem, (ii) avoid or minimise stress, includes coping strategies that have a common function of avoiding or minimising the stress, (iii) depend on seeking others for support, which involves other people as resources, either for emotional support or for direct assistance and (iv) involve withdrawal and helplessness includes strategies of escaping or becoming helpless and doing nothing (Ayers et al., 1996; Zimmer-Gembeck & Skinner, 2008a, 2008b; Zimmer-Gembeck & Locke, 2007).

The literature states that refugees use several coping strategies after exposure to stressful life situations. Some coping strategies were considered effective, whereas others were ineffective (Alzoubi et al., 2019; Khamis, 2021). Some effective coping strategies identified in the literature are problem-solving, cognitive reframing or finding meaning in the situation, having hope for the future, social support, talking with peers and religious practices (Al-Smadi et al., 2017; Braun-Lewensohn et al., 2009; Gladden, 2012). Ineffective coping strategies identified are avoidant coping, withdrawal, distraction, substance or drug abuse, disconnection, or separation (Finklestein et al., 2012; Seglem et al., 2014).

Coping strategies can play a protective role by buffering the negative effects of stress on health and well-being(Compas et al., 2001). Research also suggests that how one copes with stress is associated with one's mental health and well-being (Cheng et al., 2014; Evans & Kim, 2013). Several studies have been conducted on youth refugees'coping strategies. For instance, a study conducted in the Eastern Democratic Republic of Congo among conflict-affected youth found that cognitive and behavioural coping strategies were mutually reinforced after exposure to different traumatic experiences (Cherewick et al., 2015). Another study conducted among Sudanese refugees also reported that refugees used several coping strategies across all phases of migration, including reliance on religious beliefs, cognitive strategies and social support (Khawaja et al., 2008).

A comparative study of non-refugee Ugandan and Sudanese refugee children living in camps found that adolescent refugees employ emotion-focused coping strategies more frequently than problem-focused ones (Paardekooper et al., 1999). Paardekooper et al. (1999) attributed reliance on emotion-focused strategies in refugee camps to several constraints and few opportunities for children to implement problem-focused strategies. Another study of adolescents in the United States found that exposure to nearly all forms of traumatic experience was strongly associated with increased negative emotion-focused coping but unrelated to problem-focused and positive emotion-focused coping behaviours (Rachel et al., 2018). Dumont and Provost (1999) suggest that adolescents and adults perceive the severity of stressors differently. For example, youth are more likely to be frustrated by frequent daily problems. In contrast, adults can differentiate between minor stressors that can be easily managed and major stressors that require more attention.

While previous studies have explored coping strategies among refugee youth, gaps remain in understanding how adolescent refugees specifically cope with trauma across different migration phases. Adolescence is a unique developmental stage characterised as a period of rapid growth and development concerning physical, mental, social, emotional, sexual and other aspects of development. Adolescent refugees are especially vulnerable to traumatic events due to their incomplete bio-psychosocial and cognitive development, dependence, and underdeveloped coping skills (Lau & Thomas, 2008). There is lack of evidence to draw specific conclusion about adolescent. Adolescents are defined in this study as individuals aged 12 to 18 coping with trauma related to forced migration. Additionally, the extent to which different coping strategies influence long-term adjustment is unclear. Thus, there is a need to summaries research evidence and offer policy direction about adolescent refugees. This scoping review aims to systematically examine the scope and nature of evidence on adolescent refugee coping strategies. Specifically, it seeks to (1) identify the types of coping strategies adolescent refugees employ, (2) explore the relationship between traumatic exposure and coping strategies, and (3) determine which coping strategies facilitate or hinder adjustment to a new environment.

## Methods

The PRISMA- Extension for Scoping Reviews (PRISMA-ScR) is a reporting guideline for scoping reviews. This reporting guideline contains 20 essential reporting items and two optional items to be included when conducting a scoping review (Tricco et al., 2018). We adopted the PRISMA Extension for Scoping Reviews (PRISMA-ScR) statements to report the results using 20 essential items in this scoping review.

### Eligibility

Articles had to be peer-reviewed publications based on primary data to be included in this review. Articles were published between January 1, 2001, and June 20, 2021—a period marked by significant refugee crises caused by conflicts, political instability, and humanitarian disasters. Major events during this time included the wars in Afghanistan and Iraq, conflicts in South Sudan and the Democratic Republic of Congo, and the Syrian and Rohingya crises. Additionally, only articles written in English were considered. Studies of all designs were included, provided they met the following inclusion criteria:

(i) focused on the relationship between trauma and coping strategies was explicitly examined and discussed;
(ii) trauma was the primary predictor variable and the main focus of the study;
(iii) coping strategies were analysed as outcome variables;
(iv) focused on adolescent refugees and/or asylum seekers aged 12–18 years as participants, including all genders; and
(v) articles were written in English.

Studies were excluded from the review if studies:

(i) did not specify the association between traumatic experience and coping strategies,
(ii) did not include adolescent refugees or asylum seekers,
(iii) were systematic or scoping reviews of literature,
(iv) were published before January 1, 2001, and after June 20, 2021. Also, studies not published in peer-reviewed journals (e.g., dissertations, conference proceedings, book chapters, or reports).

### Search Strategy

Four electronic databases were selected to ensure comprehensive coverage of health, psychological, and social literature relevant to adolescent refugee coping {Ebsco Host offers access to multiple specialised databases (Academic Search Complete, CINAHL Plus with Full Text, SoINDEX, Health Source: Nursing/Academic Edition, Medline and Psyc ARTICLES), while PubMed offers access to biomedical and mental health research, WoS and SCOPUS were included due to interdisciplinary research and citation tracking access}. Articles were searched between February 3, 2021, and June 28, 2021, to identify relevant peer-reviewed articles. Reference lists of the included articles were also searched for additional information.

The search included the following terms and related concepts alone and in various combinations:

"trauma OR traumatic event OR exposure OR experience OR war trauma OR torture"AND"coping OR coping strategy OR response OR mechanism OR style OR skill"AND"adolescent OR youth OR teen"AND"refugee OR asylum-seeker".

### Data Extraction

Data were extracted using Covidence software and exported into an excel spreadsheet form. We extracted the following data on articles: primary author and year of publication, source country, study design, participant characteristics, sample size and sampling technique and results based on the inclusion criteria. Two reviewers (SD & AB) searched all the potential articles from the selected databases. They independently screened all the potentially relevant titles and abstracts of the articles identified through search strategies. Another two reviewers read the full texts and extracted the data independently (SD & EK). Uncertainty was resolved via discussion with one of the reviewers (NR). Finally, another reviewer (CJ) verified all the data presented in Tables 1 and 2.

**Table 1:** Description of included studies

Authors (years)	Country	Study design	Participant Characteristics and origin	Sample size and sampling technique
Goodman (2004)	United States	Case study	Sudanese unaccompanied refugees, Fourteen male participants Aged between 16 to 18 years	N = 14 Non-random sampling
Halcón et al. (2004)	United States	Cross-sectional	Somalia and Ethiopia origin participants, 207 (61.2%) were male, and 131(38.8%) were female participants Aged between 18 to 25 years	N = 338 Varieties of non-random sampling techniques, including targeted, linkage and snowball sampling
Khamis (2021)	Lebanon and Jordan	Cross-sectional	Syrian refugee school-age children and adolescents, 461 (46.1%) were males, and 539 (53.9%) were females aged 7 to 18 years (M = 11.30; standard SD = 2.65)	N = 1000 Non-random sampling
McGregor et al. (2015)	Australia	Cross-sectional	Participants originated from Burma, Sudan, Kosovo, Afghanistan, Pakistan, Guinea, Tanzania, Uganda, Nepal, Syria, Ethiopia, Kenya, Rwanda, Congo, Iran, and Bhutan 31 female and 19 male Aged between 12 and 21 (M = 16.63, SD = 2.51)	N = 50 Convenience and snowball sampling
Ryu (2020)	South Korea	Cross-sectional	Samples were originated from North Korea The sample comprised 81 male adolescents (40.1%) and 121 female adolescents (59.9%) The average age was 17.68 years, ranging from a minimum of 13 to a maximum of 24 years	N = 202 Convenience sampling

**Table 2:** Results on types of coping strategies and the relationship between traumatic experience coping strategies

Authors	Types of coping strategies	Relationship between trauma and coping strategies
Goodman (2004)	Fou Four themes of coping strategies were identified: (i) collectivity and communal self, (ii) suppression and distraction, (iii) making meaning, and (iv) hopelessness to hope () 9	Feelings of collectivity and community provided strong protection against the traumas and hardships experienced Suppression and distraction were the main psychological coping strategies to cope with the trauma they experienced The participants found meaning in their cultural and religious beliefs regarding suffering and life Hoping for and planning for the future became a major impetus for survival and helped participants endure the hardship and boredom of the refugee camps
Halcón et al. (2004)	Different types of specific coping strategies were identified, such as prayer, sleep read, talk about problems with friends, watch TV, exercise, go to clubs, go to work see the doctor, take medicine	Male and female youth differed in their strategies for coping with the traumatic experience. Young women were more likely to talk about their problems with friends (45% Versus. 17%, p <.01), whereas young men were more likely to cope by exercising (14% Versus. 5%, p <.01)
Khamis (2021)	Two categories of coping strategies: Positive coping styles: such as cognitive restructuring, problem-solving, emotional regulation, social support, and Negative coping styles: such as distraction, social withdrawal, criticising, self-blaming others, wishful thinking, and resignation	Avoidant coping strategies have an adverse effect on refugee children's maladjustment Emotion regulation is associated with reduced neuroticism and behavioural and emotional disorders
McGregor et al. (2015)	Active coping, Avoidant Coping, Support- seeking coping	There was no evidence of an association between coping style and family separations
Ryu (2020)	Two active coping strategies: (i) problem-focused coping and (ii) social support-focused coping	The traumatic experience was significantly correlated with social support-focused coping (β = −0.222, p <.05), whereas the relationship between trauma and problem-focused coping was not significant (β = 0.031, p <.05)

### Data Synthesis

All the included studies were grouped based on the types of information they analysed and summarised based on the descriptive characteristics of studies (primary author and year of publication, source country, study design, participant characteristics, sample size and sampling technique) and results based on the inclusion criteria. Results were also presented based on this review's objectives. Data extracted from the included studies based on were presented in tabular form.

## Results

**Fig. 1:**
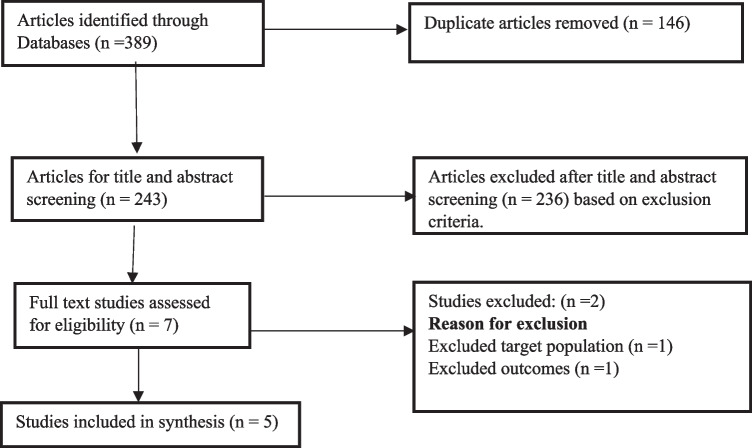
Flow diagram of the scoping review selection process

A total of 389 articles were identified as potentially relevant to the study. After the removal of duplications, 243 studies were screened. Based on titles and abstracts screening, seven articles were considered relevant for further full-text assessment and reading. Then, five articles were included in the scoping review based on the inclusion and exclusion criteria. Figure 1 presents a flow diagram for the search and screening process.

Table 1 presents the following data from the included articles: author (s), year of publication, the country where the study took place, study design, participant characteristics and country of origin, and sample size and sampling technique. Two studies were conducted in the United States (Goodman, 2004; Halcón et al., 2004), two in Asian countries (Lebanon, Jordan, and South Korea) (Khamis, 2021; Ryu, 2020) and one study from Australia (McGregor et al., 2015). Four included studies (Halcón et al., 2004; Khamis, 2021; Matheson et al., 2008; McGregor et al., 2015; Ryu, 2020) utilised a cross-sectional research design, and one study (Goodman, 2004) employed a case study; all the included studies employed forms of non-random sampling. Two studies included participants only from African countries (i.e. Somalia, Sudan, Ethiopia), and two studies included participants only from Asian countries (i.e. Syria and North Korea). One study includes participants from African and Asian countries (i.e. Burma, Sudan, Kosovo, Afghanistan, Pakistan, Guinea, Tanzania, Uganda, Nepal, Syria, Ethiopia, Kenya, Rwanda, Congo, Iran, and Bhutan). In total, 1604 participants were included across the included studies, of which more than half (n = 823) were female participants. The smallest study included 14 participants and the largest included 1000 participants.

Table 2 provides information for each included article on the types of coping strategies employed by the participants and results for the relationship between trauma and coping strategies.

## Types of Coping Strategies

All the included studies reported different types of coping strategies. For instance, Goodman (2004) identified four themes of coping strategies employed by participants after trauma exposure: (i) collectivity and communal self, (ii) suppression and distraction, (iii) making meaning, and (iv) hopelessness to hope. Halcón et al. (2004) identified specific coping strategies such as praying, sleeping, reading, talking about problems with friends, watching TV, exercising, going to clubs, working, seeing doctors, and taking medicine. Khamis (2021) identified two categories of coping used by the participants: positive coping, which includes cognitive restructuring, problem-solving, emotional regulation, social support, and negative coping styles, including distraction, social withdrawal, and criticising self-blaming others, and wishful thinking and resignation. In another study, McGregor et al. (2015) also reported active, avoidant and support-seeking coping strategies. Finally, Ryu (2020) found problem-focused and social support-focused coping strategies employed by adolescent refugees. Based on the results of the included studies, emotion-focused, avoidant and social support/support-seeking coping strategies were the most commonly observed coping strategies among the participants of the studies.

## Exploring the Relationship Between Traumatic Experiences and Coping Strategies

The findings of the included studies on the association between traumatic experiences and coping strategies were mixed. Among the included studies, one article reported no evidence was found to support a relationship between traumatic experiences and coping (McGregor et al., 2015), and four articles showed the association between these variables(Goodman, 2004; Halcón et al., 2004; Khamis, 2021; Ryu, 2020). McGregor et al. (2015), researched youth refugees in Australia and found no evidence to support the relationship between family separation as a traumatic experience and coping styles. However, emotion-focused coping strategies were strongly associated with traumatic experiences and hardships among Sudanese youth refugees (Goodman, 2004). A study by Khamis (2021) also found that avoidant coping strategies adversely affect children's refugee maladjustment. In contrast, emotional regulation is associated with reduced neuroticism and behavioural and emotional disorders. Halcón et al. (2004) found that young women were likelier to talk about their problems with friends, whereas men were more likely to cope by exercising. Ryu (2020) also reported that traumatic experience was significantly correlated with social support-focused coping; however, trauma and problem-focused coping were not significantly associated. Most of the included studies (n = 4) indicated that traumatic experiences are more strongly associated with emotion-focused, avoidant and social support coping strategies than active and problem-focused ones. This review indicated that traumatic experiences among adolescent refugees were strongly related to emotion-focused, avoidant and social support coping strategies.

## Adaptive and Maladaptive Coping

Research suggests that how one copes with stress is associated with one's mental health and well-being (Cheng et al., 2014; Evans & Kim, 2013). Among the included studies, only one article reported that avoidant coping strategies resulted in maladjustment in young refugees(Khamis, 2021). In contrast, emotional regulation is associated with reduced behavioural problems and mental health disorders (Khamis, 2021). The remaining studies do not describe adolescent refugee adaptiveness after using particular coping strategies.

## Discussion

All the included studies identified the coping strategies employed by participants after exposure to the traumatic experience. The results indicate that adolescent and youth refugees used various coping strategies after exposure to trauma. Emotion-focused, avoidant and social-support coping strategies are the most common types employed by study participants. This finding corroborates the ideas of Alzoubi et al. (2019) that refugees use several coping strategies after exposure to stressful life situations. It is also consistent with previous studies, which reported that refugees used ineffective coping strategies such as avoidant coping, withdrawal, distraction, substance or drug abuse, disconnection, or separation (Finklestein et al., 2012; Seglem et al., 2014). Moreover, this result is in line with Paardekooper et al. (1999), which found that adolescent refugees employ emotion-focused than problem-focused coping strategies. Paardekooper et al. (1999) attributed the reliance on emotion-focused strategies in the context of refugee camps may be due to several constraints and few opportunities to implement problem-focused strategies.

On the other hand, the current finding contradicts the findings of previous studies, which reported that refugees used effective coping strategies such as problem-solving, cognitive reframing, finding meaning in the situation, hope for the future, social support, talking with peers and religious practice (Al-Smadi et al., 2017; Braun-Lewensohn et al., 2009; Gladden, 2012).

The possible explanation for the dominance of emotion-focused and avoidance coping strategies may be related to the structural and contextual limitations that may refugees face. Living in uncertain, resource constrained environment such as in refugee camps or unstable host communities may limit their ability to engage in problem- focused coping. As a result, emotion-focused and avoidance coping strategies may be the only accessible route for psychological survival. On the other hand, problem-solving coping strategies could be associated with individuals with strong support systems, such as family cohesion, stable education, or access to psychological services. It is also plausible that culture and individual differences, as well as the specific nature of the trauma, have a vital mediating role in the choice of coping strategies.

This study also indicates that most studies show traumatic experiences are more strongly associated with emotion-focused, avoidant and social support coping strategies than active and problem-focused ones. This finding was consistent with previous research indicating that adolescent refugees employ emotion-focused coping strategies than problem-focused (Paardekooper et al., 1999). It is also in line with Rachel et al. (2018), which indicated that trauma was unrelated to problem-focused and positive emotion-focused coping behaviours but strongly associated with increased negative emotion-focused coping. A resealable explanation for these findings could be that unresolved trauma, especially from prolonged displacement or chronic instability, may impair executive functioning and emotional regulation, pushing individuals toward short-term emotional relief rather than long-term resolution strategies.

However, one surprising finding in this review was that one included study McGregor et al. (2015) showed no evidence between the association of family separation as a traumatic experience and coping strategies employed. The possible reason for this outcome might be the number of traumatic exposures, trauma types experienced by participants, and the small cross-sectional sample size (n = 50) of this study may affect the association between traumatic experience and coping strategies.

This review also found that avoidant coping strategies resulted in maladjustment in young refugees, whereas emotional regulation is associated with reduced behavioural problems and mental health disorders. This finding collaborated with previous findings, which identified avoidant coping as an ineffective coping strategy (Finklestein et al., 2012; Seglem et al., 2014) and emotion regulation as an effective coping strategy (Al-Smadi et al., 2017; Braun-Lewensohn et al., 2009; Gladden, 2012). In addition, this finding indicated that refugee adolescent use of avoidant coping strategies was associated with maladjustment and emotion regulation coping strategies were related to reduced mental health problems and adjustment.

## Strengths and Limitations

The strengths of this scoping review include articles searched from four electronic databases, which allowed the reviewers to get adequate articles for the study. Furthermore, most studies reported strong methods (such as large sample sizes and reliable measurements). However, the findings of this scoping review are subjected to the following limitations. First, the review did not include grey and non-English literature, resulting in the absence of pertinent research conducted in other languages. Second, it did not assess the included study's risk of bias which may affect the transparency of evidence synthesis results from each included article. Finally, the inclusion of an adult population in some articles as the sample but not distinguishing differences based on the participant's age was one of the limitations of this study.

## Recommendations

The existing literature showed that adolescent refugees used various coping strategies to respond to the demands of stressful life situations. Thus, intervention strategies should focus on using multiple effective coping strategies, such as problem-focused coping strategies like active coping, instrumental support, planning, and positive reframing to meet the needs of children and adolescent refugees as vulnerable groups in the host community to adjust to the language, cultural, and school barriers for overall growth and development. Therefore, it is also important that future research should closely examine the links between types of traumatic experiences, the number of traumatic events and coping strategies among adolescent refugees.

## Conclusion

This scoping review was conducted to identify coping strategies and examine the association between trauma and coping strategies among refugee adolescents. This review suggests that future efforts should focus on equipping adolescent refugees with problem-solving and active coping strategies while addressing their psychosocial, cultural, and educational challenges to foster resilience and positive adjustment. Thus, future primary research on adolescent refugees should consider examining the relationship between the number of traumatic experiences and categories of coping strategies on the relationship between traumatic experience and coping among adolescent refugees.

## Data Availability

All data generated and analysed are included in the manuscript.
